# Physical Activity Patterns and Risk of Type 2 Diabetes and Metabolic Syndrome in Middle-Aged and Elderly Northern Chinese Adults

**DOI:** 10.1155/2018/7198274

**Published:** 2018-08-05

**Authors:** Qian Wang, Xu Zhang, Li Fang, Qingbo Guan, Ling Gao, Qiu Li

**Affiliations:** ^1^Department of Endocrinology, Shandong Provincial Hospital Affiliated to Shandong University, Jinan, Shandong, China; ^2^Shandong Clinical Medical Center of Endocrinology and Metabolism, Jinan, Shandong, China; ^3^Institute of Endocrinology and Metabolism, Shandong Academy of Clinical Medicine, Jinan, Shandong, China; ^4^Scientific Center, Shandong Provincial Hospital Affiliated to Shandong University, Jinan, Shandong, China

## Abstract

The main aim of this study is to quantitatively describe the status of physical activity and evaluate its levels in a rural population and to investigate the association between the quantifiable physical activity and type 2 diabetes and metabolic syndrome. In total, 2076 participants aged over 40 years were included in a cross-sectional analysis. Physical activity status and the contributions of different types of activity were evaluated. The association between social behaviors and physical activities was analyzed. In addition, the impact of physical activities on type 2 diabetes mellitus and metabolic syndrome was also analyzed by logistic regression. Approximately half of the total activity in rural areas consisted of work-related activity (49.3%) followed by commuting (30.2%) and recreational activity (20.5%). In rural areas, the prevalence of physical activity levels was 28.6% for low levels, 47.3% for moderate levels, and 24.1% for high levels. Educational level showed a significant negative association with the physical activity level. Lower physical activity shows a strong and significant association with type 2 diabetes and metabolic syndrome. In conclusion, insufficient physical activity among rural people over 40 years old increases the risk of type 2 diabetes and metabolic syndrome. Population-wide and individualized guidelines for physical activities especially recreational physical activities should be developed.

## 1. Introduction

Insufficient physical activity (PA) is now an important topic in healthcare education, especially in the context of noncommunicable diseases (NCDs) because of the high risk for cardiovascular and metabolic diseases [1]. The absence of PA is also considered an urgent public health problem worldwide [2–4]. Previous research showed that insufficient PA is already a global public health concern and is increasing rapidly especially in low-income countries.

The prevalence and incidence of diabetes mellitus (DM) and metabolic syndrome (MS) are increasing rapidly worldwide, including in China. In China, the prevalence of DM and MS was 11.6% and 33.9%, respectively, in 2010 [5, 6]. Similarly, the development of DM in rural areas of China has sharply increased from less than 1% in 1980 to 10.3% in 2010 [5, 7]. In addition, type 2 diabetes (T2DM) is the predominant form of DM [8]. Although urbanization is speeding up in recent years, the rural Chinese population is still very large. Prior to 2015, 44% of the population lived in rural areas of China. Therefore, the prevention and control of T2DM and MS are urgent issues in rural areas.

Although drug therapies have been shown to be effective in the treatment of these diseases, these therapies are expensive and may have side effects. In contrast, adopting a healthy lifestyle has become one of the major approaches to easing the burden of glucose and lipid metabolic disorders. In a cross-sectional population study that controlled for body mass index (BMI) and fat distribution, PA was negatively associated with the mean of the fasting and postload insulin concentrations in two populations at risk for diabetes [9]. Other studies also showed an important role of supervised exercise interventions in lowering glycemic and lipid profile [10–12]. In the recent years, it has become essential to update the reports to including updated PA data in light of the relationship between PA and NCDs and the increasing prevalence of T2DM and MS.

The human lifestyle has changed dramatically around the world as rapid economic development has occurred. Research showed that the prevalence of low PA among individuals aged 15 or older varied from 2.6% to 62.3% [1]. The available data indicated that low PA is increasing rapidly in developing countries. For the past few years, PA levels have been assessed using the Global PA Questionnaire (GPAQ) that is used for many epidemiologic studies of DM and cardiovascular outcomes worldwide [5, 13]. However, few studies in China have assessed PA quantitatively with a worldwide activity questionnaire. The main aim of this study is to describe the status of PA in a rural Chinese population and to review the epidemiological evidence regarding the association between insufficient PA and social or morbidity factors such as T2DM and MS.

## 2. Methods

### 2.1. Study Population

The data are from the REACTION study, which aimed to investigate the epidemiology of metabolic diseases in China [14]. All of the eligible participants were over 40 years old and had been living at their current residence in each community or village for at least 5 years. The study protocol was authorized by the ethics committee of Shanghai Jiao Tong University [14]. All participants signed the informed consent before the examination.

### 2.2. Data Collection

Data collection was performed at local health stations by a trained medical staff. A well-established questionnaire was administered by trained interviewers through a face-to-face survey. A current smoker was defined as someone who smoked more than 100 cigarettes in the lifetime and who was currently smoking cigarettes at the time of the survey. Current drinking was defined as alcohol intake more than once a month for the past 12 months. Weight was measured in kilograms while height was measured in centimeters. Waist circumference (WC) was measured at the umbilical level with the participants in the standing position. BMI was calculated as weight (kg) divided by squared height (m^2^). Blood pressure was measured at the nondominant arm three times in succession with a 3 min interval between the measurements, which was performed in a sitting position. The three readings were then averaged for data analysis. Each participant with no history of DM underwent an oral glucose tolerance test. Blood samples were collected from all participants after an overnight fast of at least 10 hours. DM- and MS-related parameters were determined. Homeostasis model assessment-insulin resistance (HOMA-IR) was estimated as fasting insulin concentration (IU/mL) × fasting plasma glucose (mmol/L)/22.5, and homeostasis model assessment of *β*-cell function (HOMA-*β*) was evaluated as fasting insulin concentration (IU/mL) × 20/(fasting plasma glucose (mmol/L) − 3.5).

### 2.3. Measurement of PA

PA data were collected through a face-to-face interview by using GPAQ. GPAQ was created by the World Health Organization to monitor PA and is used to measure PA levels. The questionnaire contains 16 questions about a typical or usual week of behavior [15]. PA was divided into three domains (work, transportation, and leisure time), and the frequency (days) and duration (minutes/hours) of moderate and vigorous intensities of PA and of sedentary behavior were recorded [15]; this information can be used to convert PA data to energy expenditure. Metabolic equivalent tasks (METs) are commonly used for quantifying PA. In addition, the three domains of PA are defined as working PA, transportation PA, and leisure time PA (LTPA). One MET is defined as the basic energy cost of sitting quietly (equivalent to a caloric consumption of 1 kcal/kg/hour) [15]. A person's caloric consumption at a moderate PA level is four times as much as that at the sitting level, and the consumption at vigorous level is eight times as much as that at the sitting level. The total activity is computed as the sum of all levels of PA performed in the three domains [15]. The normal PA level of each participant was classified as low, moderate, or high. The criteria for estimating the level are listed below. High-level classification is meeting any of the following criteria: (a) at least 25 MET hours/week of activity at a vigorous level for at least three days or (b) at least 50 MET hours/week of activity with any combination of levels for seven or more days. Moderate-level classification is meeting any of the following criteria and not meeting the high-level criteria: (a) PA at a vigorous level for at least 20 minutes per day for at least three days, (b) PA at a moderate level for at least 30 minutes for at least three days, or (c) PA for at least 10 MET hours/week of any combination for at least five days. Low-level classification is not meeting any of the abovementioned criteria in this category.

### 2.4. Definitions of T2DM and MS

According to the Chinese guideline for T2DM [16], a person was diagnosed with T2DM if the person met one or more of the following criteria: (1) typical symptoms of T2DM and a random plasma glucose level of 11.1 mmol/L or higher, (2) a fasting plasma glucose level of 7.0 mmol/L or higher, or (3) a 2-hour plasma glucose level of 11.1 mmol/L or higher. In addition, participants diagnosed with type 1 diabetes or other types of DM were excluded. If no typical symptoms were shown, the test should be repeated on another day. MS was diagnosed if three or more of the following criteria were met [16]: (1) abdominal obesity, WC ≥ 90 cm for male participants or WC ≥ 85 cm for female participants; (2) hyperglycemia, a fasting plasma glucose level of 6.1 mmol/L or higher or a 2 h plasma glucose level of 7.8 mmol/L or higher, with a diagnosis of T2DM; (3) hypertension, blood pressure of 130/85 or higher, with a diagnosis of hypertension; (4) high triglyceride, fasting plasma triglyceride of 1.7 mmol/L or higher; or (5) high-density lipoprotein cholesterol (HDL-C), fasting plasma HDL-C lower than 1.04 mmol/L. Overweight was defined as a BMI of 24.0 to 27.9, and obesity was defined as a BMI of 28.0 or higher [16].

### 2.5. Definitions of High BMI, High HOMA-IR, and Low HOMA-*β*

The definition of high BMI was 25.0 kg/m^2^ or higher and was based on the China Diabetes Guideline [17]. We close the other cut-off points at the 50th percentiles because of the lack of definitive criteria for HOMA-IR and HOMA-*β* for the Chinese population.

### 2.6. Data Analysis

PA and different types of PA were evaluated in the overall population and in the subgroups according to sex, age, BMI, WC, smoking, drinking, and education. Differences between mean values were tested using Student's *t*-test. The chi-squared test was used to analyze whether there were any statistically significant differences in the prevalence of the various PA levels for the different stratifications. Logistic regression models were used to analyze the association between social behaviors and PA. Adjusted odds ratios (OR) were calculated with 95% confidence intervals (95% CIs). The association between PA types and T2DM or MS was also analyzed with logistic regression. The relationships between PA levels and some related parameters such as BMI, HOMA-IR, and HOMA-*β* were also analyzed with logistic regression.

## 3. Result

### 3.1. Sociodemographic Characteristics

The basic characteristics of the total subject population (*n* = 2076, mean age 55.2 ± 8.5 years) are presented in Table 1. Age, WC, triglyceride, HDL-C, and 2-hour plasma glucose were significantly different between men and women (*p* < 0.05). In addition, men accounted for 47.2% of the total participants, while women account for 52.8%. Among the total subject population, the prevalence of T2DM and MS was 26.0% and 41.2%, respectively. There were significant differences in the prevalence of both T2DM (*p* = 0.035) and MS (*p* < 0.001) when the participants were grouped by sex.

### 3.2. PA Status and Types of PA

Total PA, working PA, LTPA, transportation PA, and the contributions of each domain are shown in Table 2. Working and transportation PAs were the major components of the total PA. Approximately half of the total activity was contributed by working PA (49.3%) followed by transportation PA (30.2%) and LTPA (20.5%). Generally, with increases in age, educational level, and WC, total PA tended to decrease. No trend in total PA was found when the participants were grouped by BMI.

### 3.3. Prevalence of PA Levels (Low, Moderate, and High)

According to the classification criteria, the prevalence of PA levels in the overall papulation was 28.6% for low levels, 47.3% for moderate levels, and 24.1% for high levels (Table 3). The prevalence of low PA was higher in men (29.4%) than in women (27.8%), but the difference was not significant. No trends in prevalence were found when we classified the prevalence of the various PA levels by BMI. The prevalence of a high PA level decreased as the educational level increased (*p* < 0.001).

### 3.4. Associations between Total PA and Educational Levels, Smoking, and Drinking

As presented in Table 4, the logistic regression model showed a significant negative association between the educational level and PA levels (low and moderate levels versus a high level) after adjusting for age and sex. The OR (95% CI) across decreasing categories of educational levels was 1.00 (reference), 2.53 (1.49–4.30), 6.94 (4.12–11.68), 8.89 (5.13–15.40), and 8.54 (4.70–15.52) (*p* < 0.001 for trend). No significant correlation was observed between smoking or drinking and the PA level. Same results were showed in the univariate logistic regression between PA and smoking/drinking status when we stratified the population based on sex and age (Supplement Table 1).

### 3.5. Insufficient PA Is a High Risk Factor for T2DM, MS, and Metabolic Parameters

Furthermore, we examined whether the level of PA has a significant association with T2DM or MS (Table 5). After adjustment for age and sex, the moderate PA level showed a significant association with T2DM, MS, and WC. In addition, the low level of PA showed a significant association with MS and WC but not with T2DM. With respect to metabolic parameters, participants with higher PA exhibited lower levels of WC, diastolic blood pressure, triglyceride, and fasting plasma glucose values but higher levels of HDL-C. When we analyzed the relationship between PA levels and some metabolic parameters, PA levels only showed significant association with HOMA-IR with and without adjusting for age and sex (Table 6). We also found that PA levels could be grouped according to the presence of T2DM or MS.

## 4. Discussion

A few studies of urban Chinese adults were performed in recent years, while no quantitative PA study has been performed. In addition, most of these studies focused on LTPA. However, among Chinese adults, the participation in LTPA was rather low in the early 2000s, while nonleisure time activities, including activities related to housework, commuting, or work, were the main sources of daily PA. In this study, men were generally shown to spend less time in working PAs than women. The results of this study also showed that working PA is the major component of total PA for our participants. These results were consistent with those in many developing countries where working PA, in contrast to LTPA, contributes to half or more of the overall PA [18, 19]. The existence of less industrialization and fewer lifestyle changes and the greater engagement in vigorous-intensity activities such as agricultural work in the rural areas of developing areas might also be factors that determine the contribution of different types of PA.

In our research, 28.6% of the population had a low PA level (Table 3). Among several studies, the proportion varies from 35.4% in one study [20] to 49.2% in another [21], depending on the different social conditions and the different approaches for selecting levels. A review demonstrated that PA estimates varied markedly even within a single country when studies were performed using different surveys in similar time periods [22]. In general, the results showed that older people and more highly educated people are less likely to have a high level of PA. A low PA level is more common in men than in women, which is consistent with the results of other studies [22, 23]. Because the data were based on a questionnaire, the standard of activity intensity for the questionnaire was based on relative cardiopulmonary capacities, which differ between sexes. In contrast, in our research, BMI showed no association with the PA level, which was consistent with the previous studies [24]. Few of these studies focused on the correlation between social behavior and PA levels. Educational levels had an obvious negative correlation with PA levels after adjusting for age and sex, consistent with previous studies [7, 9]. Nevertheless, smoking or drinking did not show a significant association with PA in our study. Although our research found no significant correlation between smoking or drinking and PA, we should be cautious about recommending alcohol and cigarette consumption for overall human health because a strong association between smoking or drinking and PA was found in other studies.

With their increasing incidence and prevalence, T2DM and MS are gaining increasing attention in China and the prevalence of DM and MS in adults was 11.6% and 35%, respectively, in China in 2010 [5, 6]. However, in our study, the prevalence of T2DM and MS was 26.01% and 41.23%, respectively, in 2011. The high prevalence in our study may be related to the age of participants, who were selected to be more than 40 years old. Currently, endocrinologists in China are putting more effort into prevention and they now strongly support its implementation. As recommended by the ADA, PA is one of the key components in the management of T2DM and MS. Previous studies demonstrated that regular increased PA can effectively improve blood glucose control and reduce the complications of T2DM [10, 25]. Other studies have shown that DM can benefit from even a small level of PA that is even less than the suggested minimum dose that is recommended in guidelines [26, 27]. Therefore, exercise is undoubtedly viewed as an important component in the treatment of DM [28]. Although several studies conducted in developed countries reported a negative association between PA and T2DM or MS, few quantitative studies of PA and its association with T2DM and MS have been performed in China. In view of the high prevalence of T2DM and MS in rural China and the lack of knowledge about T2DM or MS prevention, it is necessary to quantify PA and determine the association between PA and T2DM or MS. This analysis showed that PA was significantly and inversely associated with the risk of developing T2DM and MS. A significant correlation was shown even with each item of MS except for hypertension. The lack of a significant association between PA and hypertension is consistent with the early research in China [29]. With economic development and the popularity of mechanization in China, physical labor may be decreasing. In addition, in view of the smaller proportion of LTPA relative to the overall PA, it is urgent to popularize and emphasize the importance of PA to rural people. Considering the growing burden of metabolic diseases in our country, promoting LTPA should be a strategy implemented at the national, community, and individual levels to combat the growing prevalence of metabolic diseases. BMI used to be one of the criteria for MS. The latest guidelines in China removed BMI as a criterion for MS based on the characteristics of the Chinese population. In fact, according to the DM guideline in China [16], Chinese people are less obese and the tendency for body fat to accumulate in the abdominal cavity is higher in Chinese people than in Caucasians, making it more likely for Chinese people to develop abdominal obesity. Even within people with normal body weight (BMI < 25 kg/m^2^), 14% reported a severe accumulation of intra-abdominal fat [30]. This finding may explain the nonsignificant association between PA levels and BMI. Based on our results, insufficient PA is a risk factor for insulin resistance. This finding implies that insufficient PA may increase the prevalence of diabetes by increasing insulin resistance. In addition, given our limited data, we did not delve into the mechanisms underlying insulin resistance. WHO recommends at least 150 min of moderate activity or at least 75 minutes of vigorous-intensity aerobic physical activity throughout a week. Participants in studies met the recommendation lowered not only risk of mortality but also risk of cardiovascular diseases [31, 32]. More research could be conducted to determine the association between long-term physical activity intervention and DM or MS in the future.

The main strength of the study is that it is a large population-based study performed in a rural region. The diagnosis of T2DM and MS in our study was strictly based on the criteria in the guideline for T2DM and MS in China. In addition, the quantification of PA was based on GPAQ, which has been widely used in recent studies in developing countries.

A few limitations still apply to our study. The population included in our study was from the REACTION study [14]. One of the eligibility criteria for enrollment was being aged 40–75 years, which makes the population relatively old. As a result, obesity may be more common in the population in our study than in the whole adult population; thus, our research may not reflect the exact relationship between the PA level and metabolic diseases in the adult population. Although a variety of confounders were considered, unknown residual confounding factors cannot be excluded. However, an accurate measurement of daily energy expenditure by the doubly labelled water method or motion sensors is still challenging in large population studies because these assessments are expensive and cumbersome. Since T2DM results from chronic low-grade inflammation and preexisting insulin resistance, a cohort study may be more acceptable for demonstrating the close relationship between insufficient PA and T2DM. To probe further relationships, more indexes of prediabetes, such as hsCRP, should be tested in the further research.

## 5. Conclusions

In summary, insufficient PA is highly prevalent among the rural population over 40 years old, which increases the risk of T2DM and MS. Population-wide and individualized guidelines for PA, especially recreational physical activities, should be developed.

## Figures and Tables

**Table 1:** General characteristics of the participants.

	Overall (*n* = 2076)	Men (*n* = 980)	Women (*n* = 1096)	*p* ^†^
Age (year)	55.1 ± 8.5	56.1 ± 8.5	54.3 ± 8.4	<0.001
WC (cm)	88.6 ± 10.1	90.6 ± 9.9	86.8 ± 9.9	<0.001
BMI (kg/m^2^)	25.3 ± 3.6	25.2 ± 3.6	25.5 ± 3.7	0.846
Systolic blood pressure (mmHg)	137.4 ± 20.4	140.3 ± 19.5	134.8 ± 20.7	<0.001
TG (mmol/L)	1.5 ± 1.2	1.6 ± 1.4	1.4 ± 1.1	<0.001
LDL-C (mmol/L)	2.9 ± 0.9	2.9 ± 0.9	2.9 ± 0.8	0.800
HDL-C (mmol/L)	1.4 ± 0.4	1.3 ± 0.4	1.4 ± 0.3	<0.001
TC (mmol/L)	4.9 ± 1.1	4.9 ± 1.2	4.9 ± 1.1	0.607
Fasting plasma glucose (mmol/L)	6.4 ± 2.0	6.5 ± 1.9	6.4 ± 2.1	0.175
2-Hour plasma glucose (mmol/L)	9.7 ± 4.7	10.0 ± 4.8	9.4 ± 4.6	0.013
Current smoker, %	16.3	31.2	0.9	<0.001
Former smoker, %	15.7	29.4	3.4	<0.001
Current drinker, %	18.4	36.8	1.8	<0.001
Former drinker, %	21.0	29.3	13.6	<0.001
T2DM, %	26.0	28.2	24.1	0.035
MS, %	41.2	45.7	37.2	<0.001

Data are expressed as mean ± standard deviation or as percentage. ^†^ *p* were analyzed between the variables of men and women. WC: waist circumference; BMI: body mass index; HDL-C: high-density lipoprotein cholesterol; LDL-C: low-density lipoprotein cholesterol; TC: total cholesterol; TG: triglyceride; *n*: number of subjects.

**Table 2:** The physical activity levels in different domains of the participants grouped by different factors.

Variables	Total (MET h/wk)	Working (MET h/wk)	Transportation (MET h/wk)	LTPA (MET h/wk)
*Total*	62.49	30.80	18.87	12.82
Sex				
Male	61.06	29.22	17.85	13.99
Female	63.78	32.21	19.80	11.77
Age				
40–49	67.88	31.45	19.89	16.54
50–59	67.19	35.04	21.52	10.65
60–69	52.36	25.85	15.20	11.32
70+	51.02	21.96	13.19	15.87
BMI				
−24	66.49	36.17	18.84	11.49
24–28	58.34	25.61	18.73	14.00
28+	63.42	31.24	19.24	12.93
WC				
Female < 85	67.63	35.46	18.09	14.08
Male < 90				
Female ≥ 85	58.87	27.51	19.44	11.93
Male ≥ 90				
Education				
Illiteracy	71.03	37.60	20.03	13.40
Primary school	67.90	41.45	14.67	11.77
Junior high school	80.23	44.65	22.08	13.50
Senior high school	47.68	16.96	18.46	12.26
College or above	33.88	0.64	19.28	14.02

MET: metabolic equivalent task; WC: waist circumference; BMI: body mass index; LTPA: leisure time physical activity.

**Table 3:** Prevalence of physical activity levels.

Variables	Low (%)	Moderate (%)	High (%)
*Total*	28.6	47.3	24.1
Sex			
Male	29.4	47.2	23.4
Female	27.8	47.3	24.9
*p*	0.60	0.99	0.41
Age			
40–49	31.8	41.7	26.5
50–59	29.3	44.7	26.0
60–69	26.1	53.5	20.4
70+	18.5	63.9	17.6
*p*	0.016	<0.001	0.017
BMI			
−24	26.7	47.9	25.4
24–28	30.9	47.1	22.0
28+	27.4	46.3	26.2
*p*	0.14	0.86	0.15
WC			
Female < 85	27.5	45.4	27.1
Male < 90			
Female ≥ 85	29.3	48.6	22.1
Male ≥ 90			
*p*	0.36	0.16	0.009
Education			
Illiteracy	26.5	46.3	27.2
Primary school	32.7	35.9	31.3
Junior high school	24.0	44.5	31.5
Senior high school	31.0	53.7	15.7
College or above	27.7	63.6	8.6
*p*	0.018	<0.001	<0.001

WC: waist circumference; BMI: body mass index.

**Table 4:** The association between social behaviors and physical activity (high level versus the other levels).

Social behavior	OR (95% CI)	*p*
Sex	0.73 (0.54–1.005)	0.053
Age (per 10 years)	0.65 (0.57–0.75)	<0.001
Nonsmoker	1	
Former smoker	1.21 (0.86–1.70)	0.28
Current smoker	0.93 (0.65–1.39)	0.71
Nondrinker	1	
Former drinker	1.16 (0.87–1.55)	0.31
Current drinker	1.29 (0.91–1.82)	0.15
College or above	1	
Illiteracy	8.54 (4.70–15.52)	<0.001
Primary school	8.89 (5.13–15.40)	<0.001
Junior high school	6.94 (4.12–11.68)	<0.001
Senior high school	2.53 (1.49–4.30)	0.001

OR: odds ratios; CI: confidence interval.

**Table 5:** Multivariate logistic regression for PA levels and DM and MS.

	Model 1^†^						Model 2					
	Low			Moderate			Low			Moderate		
	OR	95% CI	*p*	OR	95% CI	*p*	OR	95% CI	*p*	OR	95% CI	*p*
Low HDL-C	1.43	1.05–1.95	0.025	1.32	0.96–1.82	0.092	1.42	1.04–1.93	0.028	1.30	0.95–1.80	0.11
High TG	1.34	1.04–1.73	0.023	1.12	0.86–1.46	0.40	1.33	1.03–1.71	0.028	1.11	0.85–1.45	0.44
High plasma glucose	1.35	1.08–1.69	0.01	1.28	1.02–1.62	0.034	1.40	1.12–1.74	0.003	1.31	1.04–1.65	0.020
Hypertension	1.22	0.97–1.55	0.093	0.97	0.77–1.24	0.83	1.29	1.02–1.62	0.032	1.01	0.80–1.28	0.94
High WC	1.37	1.10–1.72	0.006	1.27	1.01–1.60	0.044	1.36	1.09–1.70	0.007	1.26	1.00-1.58	0.049
The presence of MS	1.60	1.27–2.02	<0.001	1.37	1.08–1.74	0.009	1.63	1.29–2.05	<0.001	1.38	1.09–1.75	0.007
The presence of T2DM	1.34	1.03–1.75	0.031	1.34	1.02–1.76	0.035	1.46	1.13–1.90	0.005	1.38	1.05–1.81	0.02

^†^Adjusted age and sex. Model 1 and model 2 are all reference with the high level of the PA level. HDL-C: high-density lipoprotein cholesterol; WC: waist circumference; TG: triglyceride; MS: metabolic syndrome; T2DM: type 2 diabetes; OR: odds ratios; CI: confidence interval.

**Table 6:** Multivariate logistic regression for PA levels and BMI, HOMA-IR, and HOMA-*β*.

	Model 1^†^						Model 2					
	Low			Moderate			Low			Moderate		
	OR	95% CI	*p*	OR	95% CI	*p*	OR	95% CI	*p*	OR	95% CI	*p*
High BMI	1.01	0.79–1.28	0.958	1.07	0.86–1.33	0.533	1.01	0.80–1.29	0.91	1.02	0.82–1.27	0.84
High HOMA-IR	1.35	1.06–1.72	0.014	1.33	1.07–1.65	0.011	1.36	1.06–1.72	0.014	1.33	1.07–1.65	0.011
Low HOMA-*β*	0.83	0.65–1.07	0.14	0.83	0.66–1.04	0.095	0.86	0.68–1.09	0.23	0.92	0.74–1.14	0.44

^†^Adjusted age and sex. Model 1 and model 2 are all reference with the high level of the PA level. OR: odds ratios; CI: confidence interval; BMI: body mass index; HOMA-IR: homeostasis model assessment-insulin resistance; HOMA-*β*: homeostasis model assessment of *β*-cell function.

## Data Availability

The data used to support the findings of this study are included within the article.

## Abbreviations

PA: Physical activity
NCDs: Noncommunicable diseases
DM: Diabetes mellitus
T2DM: Type 2 diabetes
MS: Metabolic syndrome
WC: Waist circumference
BMI: Body mass index
HDL-C: High-density lipoprotein cholesterol
METs: Metabolic equivalent tasks
GPAQ: Global PA Questionnaire
LTPA: Leisure time PA
OR: Odds ratios
95% CIs: 95% confidence intervals
HOMA-IR: Homeostasis model assessment-insulin resistance
HOMA-*β*: Homeostasis model assessment of *β*-cell function.

## Supplementary Materials

Supplementary Materials Table 1: univariate logistic regression between social behaviors and physical activity (high level versus the other levels).
