# Supplementary material for: Physical Activity Patterns and Risk of Type 2 Diabetes and Metabolic Syndrome in Middle-Aged and Elderly Northern Chinese Adults
Source: J Diabetes Res. 2018 Aug 5;2018:7198274. doi: 10.1155/2018/7198274 (PMC6098873; doi:10.1155/2018/7198274)
Supplement: Supplementary Materials — Table 1: univariate logistic regression between social behaviors and physical activity (high level versus the other levels). [file 7198274.f1.docx]

supplement table 1. Univariate logistic regression between social behaviors and physical activity (high level vs the other levels)

|  |  | OR(95%CI) | p |
| --- | --- | --- | --- |
| male |  | 0.73(0.54-1.005) | 0.053 |
|  | Non-smoker | 1 |  |
|  | Former smoker | 1.18(0.82-1.70) | 0.37 |
|  | Current smoker | 1.15(0.80-1.63) | 0.46 |
|  | Non-drinker | 1 |  |
|  | Former drinker | 1.08(0.74-1.58) | 0.70 |
|  | Current drinker | 1.22(0.86-1.73) | 0.28 |
| female |  |  |  |
|  | Non-smoker | 1 |  |
|  | Former smoker | 1.30(0.64-2.68) | 0.47 |
|  | Current smoker | 3.08(0.89-10.73) | 0.077 |
|  | Non-drinker | 1 |  |
|  | Former drinker | 1.13(0.77-1.68) | 0.54 |
|  | Current drinker | 1.32(0.50-3.48) | 0.57 |
| 40-50 years old |  |  |  |
|  | Non-smoker | 1 |  |
|  | Former smoker | 0.85(0.48-1.50) | 0.58 |
|  | Current smoker | 0.82(0.48-1.39) | 0.46 |
|  | Non-drinker | 1 |  |
|  | Former drinker | 0.98(0.63-1.51) | 0.91 |
|  | Current drinker | 0.70(0.42-1.18) | 0.18 |
| 50-60 years old |  |  |  |
|  | Non-smoker | 1 |  |
|  | Former smoker | 1.36(0.88-2.10) | 0.17 |
|  | Current smoker | 0.98(0.64-1.50) | 0.93 |
|  | Non-drinker | 1 |  |
|  | Former drinker | 1.15(0.77-1.72) | 0.49 |
|  | Current drinker | 1.26(0.84-1.90) | 0.26 |
| 60-70 years old |  |  |  |
|  | Non-smoker | 1 |  |
|  | Former smoker | 1.13(0.67-1.90) | 0.64 |
|  | Current smoker | 1.36(0.78-2.36) | 0.28 |
|  | Non-drinker | 1 |  |
|  | Former drinker | 0.85(0.49-1.49) | 0.57 |
|  | Current drinker | 1.30(0.78-2.16) | 0.32 |
| above 70 years old |  |  |  |
|  | Non-smoker | 1 |  |
|  | Former smoker | 1.06(0.26-4.29) | 0.94 |
|  | Current smoker | 3.00(0.92-9.83) | 0.07 |
|  | Non-drinker | 1 |  |
|  | Former drinker | 0.88(0.22-3.49) | 0.86 |
|  | Current drinker | 1.67(0.46-6.06) | 0.44 |
